# Imaging Findings of Plasmacytoma of Both Breasts as a Preceding Manifestation of Multiple Myeloma

**DOI:** 10.1155/2016/6595610

**Published:** 2016-01-27

**Authors:** Young Mi Park

**Affiliations:** Department of Radiology, Busan Paik Hospital, Inje University College of Medicine, Gaegeum-dong, Busanjin-gu, Busan 633-165, Republic of Korea

## Abstract

Breast plasmacytoma is an extremely rare tumor. It can occur as a primary isolated tumor or as an extramedullary manifestation in multiple myeloma. This report describes the unusual case of a primary extramedullary plasmacytoma that progressed to multiple myeloma within 15 months in a 35-year-old woman. The patient had been initially diagnosed with a primary extramedullary plasmacytoma of the epidural soft tissue at the cervical 6-thoracic 1 spine level and the stomach. The patient had received chemotherapy and the disease had been in remission. One year later, the disease recurred, affecting both breasts, right clavicle, and orbit. Three months later, the disease had progressed to multiple myeloma. I report this case, focusing on the findings of mammography, ultrasonography, magnetic resonance imaging, and positron emission tomography of bilateral breast plasmacytoma, and provide a review of the literature.

## 1. Introduction

Plasmacytoma is malignant plasma cell proliferative disorder derived from B cells in the bone marrow [1]. Plasmacytoma can be a primary tumor or it can be secondary to disseminated multiple myeloma (MM) and may grow within the axial skeleton or soft tissue [2]. In 2003, the International Myeloma Working Group recognized three distinct groups of plasmacytoma: solitary plasmacytoma of bone, extramedullary plasmacytoma, and multiple solitary plasmacytomas that are either primary or recurrent [3]. Solitary plasmacytoma of bone is the most common form, and it occurs as a lytic lesion within the axial skeleton [4]. Primary extramedullary plasmacytoma is rare, accounting for only 4% of all plasma cell tumors, and can be either solitary or multiple [5]. Extramedullary plasmacytoma most often arises in the upper aerodigestive tract, with a predilection for the head and neck [3, 5–7]. Multiple solitary plasmacytomas occur as multiple sites of disease in soft tissue, bone, or both soft tissue and bone [3].

Breast plasmacytoma is extremely rare. It can occur as a primary isolated tumor or as an extramedullary manifestation in MM. The published data on breast plasmacytomas predominantly include case reports, most of which are focused on the clinical signs or the histopathological characteristics of breast plasmacytoma.

Here, I report the imaging features of bilateral breast plasmacytomas that eventually progressed to MM in a 35-year-old female patient.

## 2. Case Presentation

In June 2014, a 35-year-old female patient presented with multiple nontender palpable lumps in both breasts and diplopia in her left eye. Thirteen months earlier, she had been diagnosed with primary extramedullary plasmacytoma of the epidural soft tissue at the cervical 6-thoracic 1 spine (C6-T1) level and the stomach. At that time, a bone marrow biopsy had shown normocellular bone marrow. A bone marrow examination contained less than 10% (6.2%) plasma cells. There had been no evidence of bony abnormality, end-organ damage (anemia, renal failure, and hypercalcemia), and serum or urine monoclonal protein elevation. The patient had undergone 12 cycles of chemotherapy with melphalan and prednisolone with concurrent radiotherapy of the spinal region. Serial follow-up with magnetic resonance imaging (MRI) and computed tomography (CT) of the cervical spine and abdominopelvic region had shown no tumor recurrence for a year.

**Figure 1:**
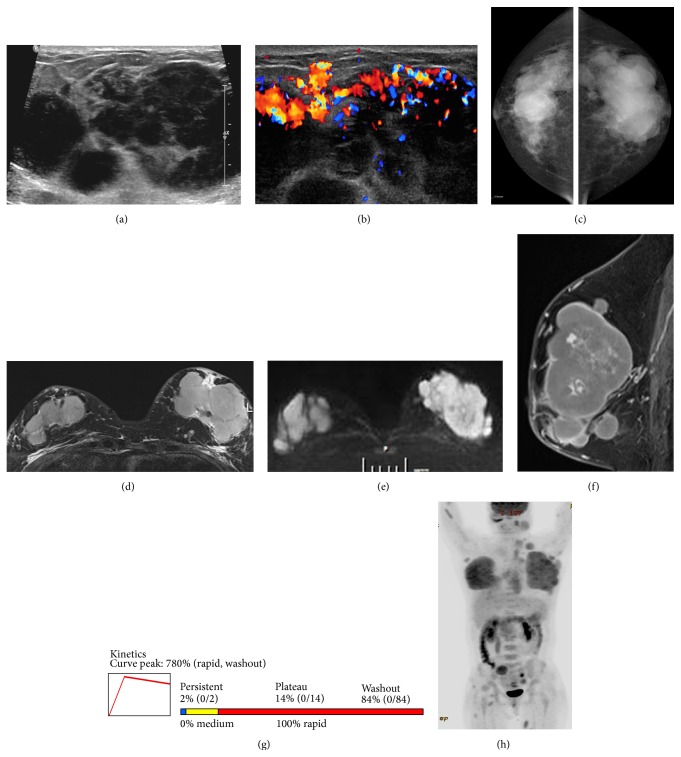
Gray scale (a) and color Doppler (b) sonograms of the breast show multiple, circumscribed, markedly hypoechoic, round or oval masses with highly increased vascularity. Mammograms (c) show circumscribed irregular conglomerated hyperdense masses invading both entire breasts. T2-weighted image of breast magnetic resonance imaging (MRI) (d) shows multiple, circumscribed, oval or round masses of high signal intensity (SI) in both breasts. The masses reveal high SI on diffusion-weighted image (e) with a low apparent diffuse coefficient value (not shown) suspicious for malignancy. Early phase of dynamic contrast-enhanced sagittal T1-weighted MRI (f) shows multiple circumscribed masses with strong homogeneous enhancement. Time-intensity curve of dynamic contrast-enhanced MRI (g) shows early strong (780% of the baseline value) and fast/delayed washout enhancement kinetics, suggestive of malignancy. Follow-up positron emission tomography-computed tomography scan (h) reveals massive breast plasmacytomas (SUVmax 6.3), invading both entire breasts. Metastatic lymphadenopathies throughout the whole body and metastatic masses in the abdomen, left cheek, both shoulders, chest wall, right buttock, and both thighs are shown.

**Figure 2:**
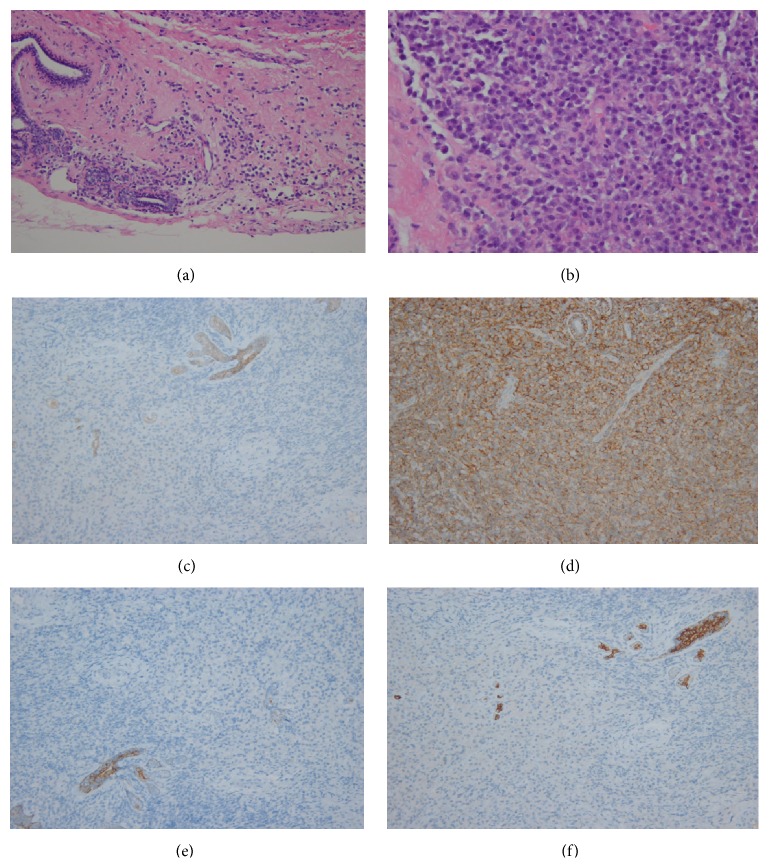
Histopathological analysis ((a) HE ×200) of left breast mass shows infiltration of plasmacytoid cells around the ductal breast tissue. The plasmacytoid cells are eccentrically located with slightly enlarged nuclei compared to mature plasma cells ((b) HE ×400). Photomicrographs show a positive reaction for CD 138 ((c) ×200), indicating plasma cell origin, and positive reactions for E-cadherin ((d) ×200), CK5/6 ((e) ×200), and CK7 ((f) ×200) in the entrapped breast ductal tissue, but negative in infiltrated cells, suggesting a nonepithelial origin.

On breast ultrasonography, multiple well-defined, oval, markedly hypoechoic masses were recognized (Figures 1(a) and 1(b)). The patient's mammograms showed multiple circumscribed or obscured, oval or round, hyperdense masses in both breasts (Figure 1(c)). Ultrasound-guided core needle biopsy of the largest mass in each breast (3.6 cm and 7.2 cm in the right and left breasts, resp.) revealed plasma cell myeloma (Figure 2). Breast MRI showed multiple, well-defined, oval or round, strongly enhanced masses with iso/high signal intensity (SI) on T1/T2-weighted images in both breasts (Figure 1(d)). The masses were characterized as high SI on diffusion-weighted imaging (DWI) with a low apparent diffusion coefficient (ADC) value, indicating diffusion restriction (Figure 1(e)). Dynamic contrast-enhanced MRI revealed massive, almost homogeneous enhancement (780%) of the baseline value at the initial phase and a delayed washout kinetic curve, suggesting malignancy (Figures 1(f) and 1(g)). On the shoulder MRI, there was a soft tissue mass in and around the medial end of the right clavicle, extending into the ipsilateral pectoralis major muscle and the anterior mediastinum. Additionally, multiple enhanced soft tissue masses were detected in the para-aortic space and the prevertebral space of the first thoracic vertebral body on MRI. On positron emission tomography- (PET-) CT, increased F-18 fluorodeoxyglucose (FDG) uptake (maximum standardized uptake value [SUVmax 6.2]) in the head and body of the right humerus, as well as in both the breasts, was discovered, suggestive of humeral involvement. A 2.8 cm soft tissue mass with increased FDG uptake (SUVmax 3.3) was also noted in the inferolateral portion of the extraconal space of the left orbit. Repeated bone marrow biopsy and aspiration results revealed 40–50% bone marrow cellularity and 3.5% bone marrow plasma cells, which did not meet the diagnostic criteria of MM [8]. However, serum protein electrophoresis revealed monoclonal gammopathy (lambda light chain), and the patient was also diagnosed with anemia; her hemoglobin levels were below 10 g/dL.

On follow-up PET-CT in August 2014 (Figure 1(h)), the extent of the myeloma lesions in both breasts (SUVmax 6.3) had increased, resulting in involvement of both entire breasts. There were metastatic lymphadenopathies in the patient's left lower neck and in the axillary, anterior mediastinal, para-aortic, prevertebral, and retrocrural areas. Metastatic masses were also found in the pancreas and retroperitoneal and intraperitoneal spaces; the right middle, right lower, and left upper lobes of the lung; the left cheek; both shoulders; the chest wall; and the right buttock and both thighs. Obstructive nephropathy occurred due to involvement of the right ureter. In August 2014, the patient died of renal and pulmonary failure and severe metabolic acidosis.

## 3. Discussion

Proliferation of malignant plasma cells is a morphological manifestation of several plasma cell malignancies [5, 9]. There are five different types of plasma cell malignancies [5, 9–11]: MM, characterized by diffuse infiltration of the bone marrow; solitary plasmacytoma of bone; extramedullary plasmacytoma without bone marrow infiltration; MM with extramedullary manifestations; and plasma cell leukemia. MM is the most common manifestation of plasma cell malignancies. Primary plasmacytoma is differentiated from multiple myeloma by the absence of hypercalcemia, renal insufficiency, and anemia, a normal skeletal survey, absence of bone marrow plasmacytosis, and serum or urinary paraprotein levels of <2 g/dL [3, 6, 7, 12]. The clinical courses and prognoses of solitary plasmacytoma of bone, extramedullary plasmacytoma, multiple solitary plasmacytomas, and MM differ.

Thirty to fifty percent of extramedullary plasmacytoma cases progress to MM with a median time of 1.5–2.5 years [13]. The presenting case also showed extremely malignant and rapid progression. The patient initially started with primary extramedullary plasmacytoma in the epidural area of the C6-T1 spine and stomach. At that time, bone marrow aspiration revealed less than 10% plasma cells and there was no evidence of bony involvement or end-organ damage. There was also no elevation of serum or urine monoclonal proteins. Therefore, the diagnosis was primary extramedullary plasmacytoma rather than extraosseous MM. The patient had received chemotherapy and the disease had been in remission. After about a year, however, relapse and progression to MM occurred. The plasma cell myelomas invaded the right humerus and clavicle as well as both breasts and the orbit. Serum protein electrophoresis showed monoclonal gammopathy at this time. Following this, dissemination of the disease to nearly the entire body occurred in just 3 months.

This patient was unusual in several aspects. Firstly, she was at a relatively young age. MM is a disease of older adults; the median age at diagnosis is 66 years. Only 10%, 2%, and 0.3% of patients are younger than 50, 40, and 30 years, respectively [1, 14]. Secondly, her bone marrow examination results showed normocellular bone marrow and fewer than 10% plasma cells. Approximately 4% of patients may have fewer than 10% bone marrow plasma cells because marrow involvement may be focal, rather than diffuse [8]. Thirdly, both her breasts were entirely invaded by multiple plasmacytomas. Of the breast plasmacytoma cases that have been described, nearly half have been bilateral [15]. The presenting case, however, showed unique image findings: on the final PET-CT scan, multiple masses of myeloma had invaded both entire breasts, forming a huge conglomerated mass.

Breast plasmacytoma is extremely rare with unknown prevalence. Surov et al. [2] reported that the prevalence of breast plasmacytoma at their institution was 1.5% of all identified patients with plasmacytoma and that, in 85% of the patients, involvement of the breast was a secondary event of MM. Primary and secondary plasmacytomas do not show significant differences in radiological characteristics, and they can be misdiagnosed as primary breast carcinoma or even as a benign process [2]. It can present as hyperdense, round or oval, masses with well- or ill-defined margins on mammography. It can also be identified by diffuse infiltration, but microcalcifications are scarce [2]. On ultrasonography, breast plasmacytoma can appear as echo-poor or hypoechoic well-defined masses with hypervascularity, but mixed hypo- to hyperechoic lesions with indistinct margins can also be revealed [2]. The posterior acoustic features are also variable; they can show posterior acoustic enhancement or no acoustic transmission and even posterior acoustic shadowing [2].

The patient in this report had similar mammographic and ultrasonographic findings to the previously reported cases. She had multiple lesions in both breasts. The differential diagnosis of multiple bilateral breast masses includes both benign and malignant conditions, such as fibroadenomas, complex cysts, focal fibrosis of the breast, fat necrosis, abscesses, phyllodes tumors, metastasis, lymphoma, and synchronous breast cancers [16]. Malignant multiple bilateral breast masses are very uncommon, but radiologists should consider metastatic or hematological disease when the imaging findings are suspicious. A history of malignancy can help in the differential diagnosis [17, 18].

To date, there are three reports on the MRI findings of breast plasmacytoma [19–21]. According to a case report by Neuhaus and Hess [19], extramedullary plasmacytomas of the breast revealed low/intermediate SI on T1/T2-weighted images and massive homogeneous enhancement with early strong/delayed washout kinetics. On the other hand, another plasmacytoma reported by Kim et al. [21] showed intermediate/low SI on T1-/T2-weighted images. After venous administration of contrast medium, a breast plasmacytoma manifested as a hypervascular mass [20]. In the present case, plasmacytomas revealed high SI on DWI with low ADC values and early strong and fast/delayed washout enhancement kinetics, suggestive of malignancy. These findings suggest that MRI may be more helpful in the differential diagnosis than mammography and ultrasonography.

Extramedullary plasmacytomas can virtually involve whole organs in the body. The few reports on the radiological manifestations of plasmacytomas in other parts of the body indicate that extramedullary plasmacytomas are nonspecific and widely variable depending on the site [22]. The differential diagnosis includes extramedullary hematopoiesis, infection, amyloidoma, and a second malignancy. Generally, it is observed as homogeneous soft tissue masses on CT, with a low T2 signal without necrosis and calcification on MRI and with high FDG uptake on FDG-PET. The MRI findings represent high cellularity composed of monoclonal plasma cells [23, 24].

The International Myeloma Working Group recommends urgent MRI or CT when extramedullary involvement is suspected. FDG-PET should be also included [3, 23]. MRI, PET/CT, or CT may be used to evaluate sites of plasma cell myeloma and to monitor the response of these sites to treatment.

In conclusion, breast plasmacytoma in the present report showed multiple circumscribed, oval or round, radiodense, and markedly hypoechoic masses in both breasts. MRI showed strongly enhanced masses with iso/high SI on T1/T2- and diffusion-weighted images and initial fast/delayed washout kinetic curve, suggesting malignancy. When multiple bilateral breast masses are found, breast plasmacytoma should be considered in the differential diagnosis of breast disorders, especially in patients with history of plasma cell malignancy. MRI may be a better option in the differential diagnosis of plasmacytomas than mammography and ultrasonography, which would contribute to a more accurate diagnosis in these patients.
